# 25,26,27,28-Tetra­kis(3-bromo­benzyl­oxy)calix[4]arene

**DOI:** 10.1107/S1600536811022975

**Published:** 2011-06-18

**Authors:** Eunji Lee, Suk-Hee Moon, Tae Ho Kim, Ki-Min Park

**Affiliations:** a Department of Chemistry and Research Institute of Natural Sciences, Gyeongsang National University, Jinju 660-701, Republic of Korea; b Department of Food & Nutrition, Kyungnam College of Information and Technology, Busan 617-701, Republic of Korea

## Abstract

In the title compound, C_56_H_44_Br_4_O_4_, the calix[4]arene unit displays the 1,2-alternate conformation with crystallograpically imposed inversion symmetry. The four phen­oxy rings of the calix[4]arene unit are twisted about the mean plane defined by the four methyl­ene C atoms bridging the benzene rings, with dihedral angles of 46.73 (6) and 66.11 (5)°. The dihedral angle between adjacent phen­oxy rings is 74.75 (7)°. The two pendant bromo­phenyl rings on the same side of the calix[4]arene unit are nearly perpendicular to each other, with a dihedral angle of 72.85 (10)° due to an intra­molecular C—H⋯π inter­action. In the crystal, a Br⋯Br contact of 3.6350 (5) Å, an inter­molecular C—H⋯Br hydrogen bond and an inter­molecular C—H⋯π inter­action are observed.

## Related literature

For calix[4]arene chemistry and its applications, see: Gutsche (2008 ▶); Ikeda & Shinkai (1997 ▶). For the use of calixarenes in crystal engineering, see: Dalgrano *et al.* (2007 ▶). For the modification of calix[4]arenes, see: Asfari *et al.* (2001 ▶); Mandolini & Ungaro (2000 ▶).
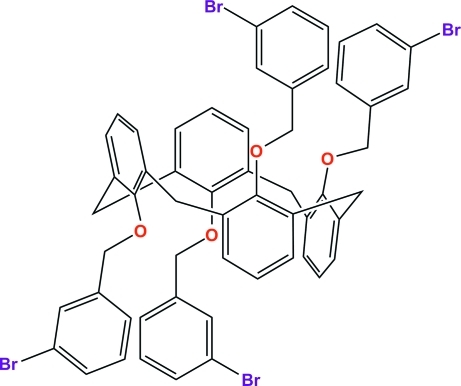


## Experimental

### 

#### Crystal data

C_56_H_44_Br_4_O_4_
*M* *_r_* = 1100.55
Monoclinic, 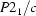

*a* = 13.4377 (8) Å
*b* = 10.4789 (7) Å
*c* = 17.1666 (12) Å
β = 103.822 (2)°
*V* = 2347.3 (3) Å^3^
*Z* = 2
Mo *K*α radiation
μ = 3.48 mm^−1^
*T* = 173 K
0.21 × 0.20 × 0.15 mm

#### Data collection

Bruker APEXII CCD diffractometer
Absorption correction: multi-scan (*SADABS*; Sheldrick, 1996) ▶ *T*_min_ = 0.529, *T*_max_ = 0.624
22506 measured reflections
5813 independent reflections
3929 reflections with *I* > 2σ(*I*)
*R*_int_ = 0.037

#### Refinement

*R*[*F* ^2^ > 2σ(*F* ^2^)] = 0.041
*wR*(*F* ^2^) = 0.106
*S* = 1.02
5813 reflections
289 parameters
H-atom parameters constrained
Δρ_max_ = 0.63 e Å^−3^
Δρ_min_ = −0.91 e Å^−3^

### 

Data collection: *APEX2* (Bruker, 2006 ▶); cell refinement: *SAINT* (Bruker, 2006 ▶); data reduction: *SAINT*; program(s) used to solve structure: *SHELXTL* (Sheldrick, 2008 ▶); program(s) used to refine structure: *SHELXTL*; molecular graphics: *SHELXTL* and *DIAMOND* (Brandenburg, 1998 ▶); software used to prepare material for publication: *SHELXTL*.

## Supplementary Material

Crystal structure: contains datablock(s) global, I. DOI: 10.1107/S1600536811022975/wn2436sup1.cif

Structure factors: contains datablock(s) I. DOI: 10.1107/S1600536811022975/wn2436Isup2.hkl

Additional supplementary materials:  crystallographic information; 3D view; checkCIF report

## Figures and Tables

**Table 1:** Hydrogen-bond geometry (Å, °) *Cg*1 and *Cg*2 are the centroids of the C23–C28 and C7–C12 rings, respectively.

*D*—H⋯*A*	*D*—H	H⋯*A*	*D*⋯*A*	*D*—H⋯*A*
C15—H15*B*⋯Br2^i^	0.99	2.91	3.652 (2)	132
C17—H17⋯*Cg*1	0.95	2.74	3.69	175
C5—H5⋯*Cg*2^ii^	0.95	2.68	3.54	151

Symmetry codes: (i) 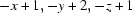; (ii) 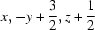.

**Fig. 1.**
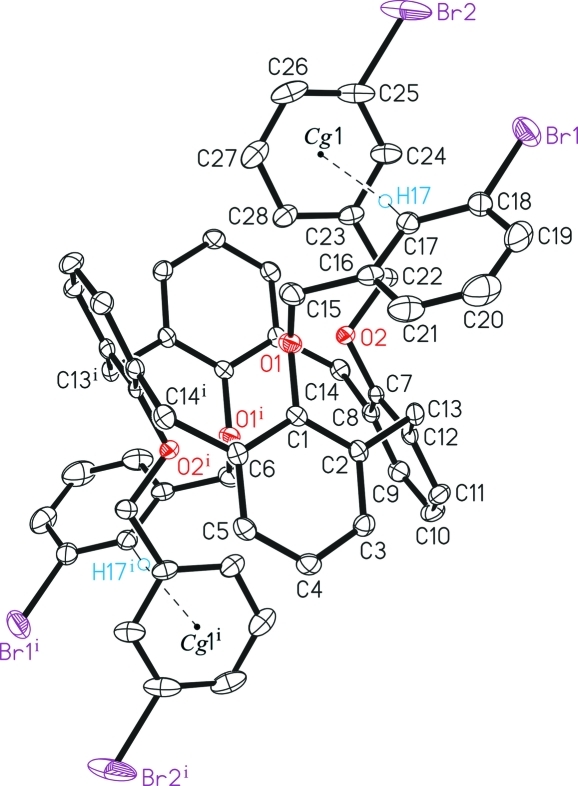
The molecular structure of the title compound with intramolecular C—H···π interactions shown as dashed lines. Displacement ellipsoids are represented at the 30% probability levels. H atoms not involved in intramolecular interactions have been omitted for clarity. H atoms are presented as a small spheres of arbitrary radius. Cg1is the centroid of the C23–C28 ring. (Symmetry code: (i) -x, -y + 2, -z + 1.)

**Fig. 2.**
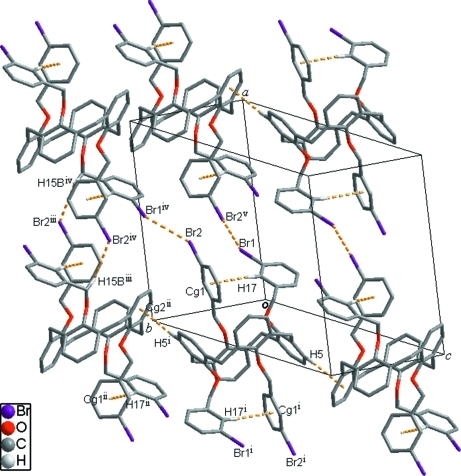
Crystal packing of the title compound with intermolecular C—H···Br hydrogen bonds, C—H···π interactions and Br···Br contacts shown as dashed lines. H atoms not involved in intra- or intermolecular interactions have been omitted for clarity. Cg1 and Cg2 are the centroids of the C23–C28 and C7–C12 rings, respectively. (Symmetry codes: (i) -x, -y + 2, -z + 1; (ii) -x, y + 1/2, -z + 1/2; (iii) x, -y + 5/2, z - 1/2; (iv) -x + 1, y + 1/2, -z + 1/2.)

## supplementary crystallographic information

### Comment

Calix[4]arenes are macrocycles employed widely in supramolecular chemistry (Gutsche, 2008; Ikeda & Shinkai, 1997) and crystal engineering (Dalgrano *et al.*, 2007) because they can be easily modified by selective reactions involving the upper and/or the lower rim of the molecule (Asfari *et al.*, 2001; Mandolini & Ungaro, 2000). Calix[4]arenes can adopt several conformations, of which the 1,2-alternate conformation is much less commonly found. For that reason, the title compound, adopting the 1,2-alternate conformation, was prepared for use as a starting material for the preparation of ligands for construction of metallo-supramolecular architectures *via* appropriate chemical modification at the bromine positions.

In the crystal structure of the title compound, as shown in Fig. 1, the calix[4] unit adopts an 1,2-alternate conformation, located on a crystallographic inversion centre. Therefore the asymmetric unit consists of one half-molecule. The four phenoxy rings of the calix[4]arene unit are twisted about the mean plane defined by the four methylene C atoms (C13, C14, C13^i^ and C14^i^) bridging the benzene rings, with dihedral angles of 46.73 (6)° for the C1–C6 ring and 66.11 (5)° for the C7–C12 ring (symmetry code: (i) -*x*, -*y* + 2, -*z* + 1). The adjacent phenoxy rings make a dihedral angle of 74.75 (7)°. The two pendant bromophenyl rings on the same side of the calix[4]arene unit are nearly perpendicular to each other, with a dihedral angle of 72.85 (10)° due to the intramolecular C—H···π interaction; *Cg*1 is the centroid of the C23–C28 ring, H17··· *Cg*1 2.74 Å and C17—H17···*Cg*1 174.5 ° (Table 1, Fig. 1).

In the crystal structure, as shown in Fig. 2, a Br···Br contact of 3.6350 (5) Å, an intermolecular C—H···Br hydrogen bond and intermolecular C—H···π interaction are observed (Table 1). These intermolecular interactions may contribute to the stabilization of the crystal packing.

### Experimental

To a refluxing suspension of calix[4]arene (2.00 g, 4.71 mmol) and caesium carbonate (7.50 g, 23.0 mmol) in dry acetone (200 ml) was added dropwise 1-bromo-3-(methylbromo)benzene (4.80 g, 19.2 mmol) in dry acetone (20 ml). The mixture was stirred and refluxed for 12 h and cooled to room temperature. The solvent was removed under reduced pressure. The residue was neutralized with 10% hydrochloric acid and extracted with dichloromethane. The organic layer was washed three times with water and dried over anhydrous magnesium sulfate. After removal of the solvent under reduced pressure, the residue was recrystallized from dichloromethane-hexane to give the title compound in 27% yield as a white solid. Single crystals suitable for X-ray diffraction were obtained by evaporation of a dichloromethane solution.

### Refinement

All H atoms were positioned geometrically and refined using a riding model, with d(C—H) = 0.95 Å for C*sp*^2^—H and 0.99 Å for methylene C—H. For all H atoms *U*_iso_(H) = 1.2*U*_eq_(C).

### Crystal data

**Table:**

C_56_H_44_Br_4_O_4_	*F*(000) = 1104
*M**_r_* = 1100.55	*D*_x_ = 1.557 Mg m^−^^3^
Monoclinic, *P*2_1_/*c*	Mo *K*α radiation, λ = 0.71073 Å
Hall symbol: -P 2ybc	Cell parameters from 5179 reflections
*a* = 13.4377 (8) Å	θ = 2.3–27.1°
*b* = 10.4789 (7) Å	µ = 3.48 mm^−^^1^
*c* = 17.1666 (12) Å	*T* = 173 K
β = 103.822 (2)°	Block, colourless
*V* = 2347.3 (3) Å^3^	0.21 × 0.20 × 0.15 mm
*Z* = 2	

### Data collection

**Table:**

Bruker APEXII CCD diffractometer	5813 independent reflections
Radiation source: fine-focus sealed tube	3929 reflections with *I* > 2σ(*I*)
graphite	*R*_int_ = 0.037
φ and ω scans	θ_max_ = 28.3°, θ_min_ = 1.6°
Absorption correction: multi-scan (*SADABS*; Sheldrick, 1996)	*h* = −17→17
*T*_min_ = 0.529, *T*_max_ = 0.624	*k* = −13→10
22506 measured reflections	*l* = −22→22

### Refinement

**Table:**

Refinement on *F*^2^	Primary atom site location: structure-invariant direct methods
Least-squares matrix: full	Secondary atom site location: difference Fourier map
*R*[*F*^2^ > 2σ(*F*^2^)] = 0.041	Hydrogen site location: inferred from neighbouring sites
*wR*(*F*^2^) = 0.106	H-atom parameters constrained
*S* = 1.02	*w* = 1/[σ^2^(*F*_o_^2^) + (0.0447*P*)^2^ + 1.723*P*] where *P* = (*F*_o_^2^ + 2*F*_c_^2^)/3
5813 reflections	(Δ/σ)_max_ = 0.001
289 parameters	Δρ_max_ = 0.63 e Å^−^^3^
0 restraints	Δρ_min_ = −0.91 e Å^−^^3^

### Special details

**Table:**

Geometry. All e.s.d.'s (except the e.s.d. in the dihedral angle between two l.s. planes) are estimated using the full covariance matrix. The cell e.s.d.'s are taken into account individually in the estimation of e.s.d.'s in distances, angles and torsion angles; correlations between e.s.d.'s in cell parameters are only used when they are defined by crystal symmetry. An approximate (isotropic) treatment of cell e.s.d.'s is used for estimating e.s.d.'s involving l.s. planes.
Refinement. Refinement of *F*^2^ against ALL reflections. The weighted *R*-factor *wR* and goodness of fit *S* are based on *F*^2^, conventional *R*-factors *R* are based on *F*, with *F* set to zero for negative *F*^2^. The threshold expression of *F*^2^ > σ(*F*^2^) is used only for calculating *R*-factors(gt) *etc*. and is not relevant to the choice of reflections for refinement. *R*-factors based on *F*^2^ are statistically about twice as large as those based on *F*, and *R*- factors based on ALL data will be even larger.

### Fractional atomic coordinates and isotropic or equivalent isotropic displacement parameters (Å^2^)

**Table:**

	*x*	*y*	*z*	*U*_iso_*/*U*_eq_	
Br1	0.41607 (3)	0.68787 (4)	0.35182 (2)	0.06529 (14)	
Br2	0.48689 (2)	1.06993 (6)	0.31500 (3)	0.0952 (2)	
O1	0.15137 (12)	0.85506 (17)	0.55860 (10)	0.0291 (4)	
O2	0.04423 (11)	0.96810 (15)	0.38948 (9)	0.0242 (3)	
C1	0.07450 (16)	0.7816 (2)	0.57737 (13)	0.0232 (5)	
C2	0.01333 (17)	0.7075 (2)	0.51741 (14)	0.0244 (5)	
C3	−0.06427 (18)	0.6351 (2)	0.53749 (15)	0.0288 (5)	
H3	−0.1049	0.5805	0.4983	0.035*	
C4	−0.08325 (19)	0.6411 (2)	0.61272 (15)	0.0310 (5)	
H4	−0.1357	0.5902	0.6254	0.037*	
C5	−0.02539 (18)	0.7217 (2)	0.66956 (14)	0.0283 (5)	
H5	−0.0405	0.7282	0.7207	0.034*	
C6	0.05422 (17)	0.7931 (2)	0.65368 (13)	0.0249 (5)	
C7	−0.04927 (16)	0.9096 (2)	0.35766 (12)	0.0222 (5)	
C8	−0.12770 (17)	0.9807 (2)	0.30901 (13)	0.0255 (5)	
C9	−0.22207 (18)	0.9204 (3)	0.28019 (15)	0.0336 (6)	
H9	−0.2775	0.9669	0.2478	0.040*	
C10	−0.23581 (19)	0.7942 (3)	0.29812 (16)	0.0388 (7)	
H10	−0.3002	0.7542	0.2776	0.047*	
C11	−0.15618 (19)	0.7258 (3)	0.34591 (15)	0.0342 (6)	
H11	−0.1662	0.6386	0.3573	0.041*	
C12	−0.06149 (17)	0.7829 (2)	0.37760 (13)	0.0245 (5)	
C13	0.02461 (18)	0.7085 (2)	0.43178 (14)	0.0266 (5)	
H13A	0.0241	0.6196	0.4121	0.032*	
H13B	0.0912	0.7474	0.4301	0.032*	
C14	−0.11303 (18)	1.1161 (2)	0.28319 (14)	0.0284 (5)	
H14A	−0.1817	1.1533	0.2609	0.034*	
H14B	−0.0774	1.1124	0.2390	0.034*	
C15	0.25431 (18)	0.8158 (3)	0.59555 (16)	0.0380 (6)	
H15A	0.2573	0.7862	0.6508	0.046*	
H15B	0.3007	0.8901	0.5991	0.046*	
C16	0.29142 (17)	0.7103 (3)	0.54979 (16)	0.0333 (6)	
C17	0.32711 (17)	0.7399 (3)	0.48246 (16)	0.0318 (6)	
H17	0.3272	0.8259	0.4650	0.038*	
C18	0.36244 (19)	0.6437 (3)	0.44102 (18)	0.0412 (7)	
C19	0.3620 (2)	0.5183 (3)	0.4642 (3)	0.0606 (10)	
H19	0.3858	0.4530	0.4348	0.073*	
C20	0.3264 (3)	0.4894 (3)	0.5308 (3)	0.0694 (11)	
H20	0.3254	0.4032	0.5477	0.083*	
C21	0.2918 (2)	0.5851 (3)	0.5734 (2)	0.0536 (8)	
H21	0.2681	0.5638	0.6196	0.064*	
C22	0.11169 (17)	0.9651 (3)	0.33740 (15)	0.0305 (5)	
H22A	0.0812	1.0128	0.2877	0.037*	
H22B	0.1226	0.8758	0.3226	0.037*	
C23	0.21228 (17)	1.0240 (2)	0.37845 (16)	0.0306 (5)	
C24	0.28954 (19)	1.0230 (3)	0.33757 (17)	0.0411 (7)	
H24	0.2776	0.9876	0.2852	0.049*	
C25	0.3838 (2)	1.0737 (3)	0.3736 (2)	0.0532 (9)	
C26	0.4038 (2)	1.1250 (3)	0.4500 (2)	0.0551 (9)	
H26	0.4693	1.1591	0.4743	0.066*	
C27	0.3268 (2)	1.1255 (3)	0.4897 (2)	0.0485 (8)	
H27	0.3396	1.1600	0.5423	0.058*	
C28	0.23048 (19)	1.0765 (2)	0.45464 (17)	0.0362 (6)	
H28	0.1775	1.0789	0.4827	0.043*	

### Atomic displacement parameters (Å^2^)

**Table:**

	*U*^11^	*U*^22^	*U*^33^	*U*^12^	*U*^13^	*U*^23^
Br1	0.04985 (19)	0.0996 (3)	0.0523 (2)	−0.00434 (18)	0.02381 (15)	−0.02819 (19)
Br2	0.03236 (17)	0.1775 (5)	0.0785 (3)	−0.0192 (2)	0.01840 (17)	0.0392 (3)
O1	0.0268 (8)	0.0341 (10)	0.0280 (9)	−0.0023 (7)	0.0100 (7)	0.0013 (7)
O2	0.0220 (7)	0.0264 (9)	0.0235 (8)	−0.0031 (6)	0.0043 (6)	−0.0022 (7)
C1	0.0246 (10)	0.0223 (13)	0.0237 (11)	0.0041 (9)	0.0081 (9)	0.0044 (9)
C2	0.0309 (11)	0.0191 (13)	0.0247 (12)	0.0048 (9)	0.0097 (9)	0.0025 (9)
C3	0.0345 (12)	0.0226 (13)	0.0292 (12)	−0.0022 (10)	0.0076 (10)	0.0014 (10)
C4	0.0358 (12)	0.0264 (14)	0.0329 (13)	−0.0022 (10)	0.0126 (10)	0.0073 (11)
C5	0.0359 (12)	0.0284 (14)	0.0230 (12)	0.0035 (10)	0.0116 (10)	0.0070 (10)
C6	0.0299 (11)	0.0228 (13)	0.0219 (11)	0.0052 (9)	0.0061 (9)	0.0036 (9)
C7	0.0232 (10)	0.0287 (13)	0.0153 (10)	−0.0039 (9)	0.0058 (8)	−0.0054 (9)
C8	0.0268 (11)	0.0317 (14)	0.0178 (11)	−0.0006 (10)	0.0052 (9)	−0.0031 (10)
C9	0.0273 (11)	0.0467 (18)	0.0246 (12)	−0.0027 (11)	0.0018 (9)	−0.0032 (11)
C10	0.0302 (12)	0.0511 (19)	0.0322 (14)	−0.0151 (12)	0.0019 (10)	−0.0062 (13)
C11	0.0396 (13)	0.0346 (16)	0.0288 (13)	−0.0142 (11)	0.0090 (11)	−0.0038 (11)
C12	0.0310 (11)	0.0281 (14)	0.0157 (10)	−0.0026 (9)	0.0083 (9)	−0.0040 (9)
C13	0.0345 (12)	0.0227 (13)	0.0241 (12)	0.0012 (10)	0.0100 (9)	−0.0025 (10)
C14	0.0321 (11)	0.0333 (15)	0.0190 (11)	0.0037 (10)	0.0043 (9)	0.0037 (10)
C15	0.0241 (11)	0.0573 (19)	0.0321 (14)	−0.0014 (12)	0.0055 (10)	−0.0067 (13)
C16	0.0216 (11)	0.0373 (16)	0.0398 (14)	−0.0032 (10)	0.0048 (10)	0.0015 (12)
C17	0.0255 (11)	0.0316 (15)	0.0383 (14)	0.0007 (10)	0.0074 (10)	−0.0034 (11)
C18	0.0263 (12)	0.0485 (19)	0.0482 (17)	−0.0011 (12)	0.0075 (11)	−0.0144 (14)
C19	0.0419 (16)	0.036 (2)	0.103 (3)	0.0035 (14)	0.0153 (18)	−0.0216 (19)
C20	0.059 (2)	0.034 (2)	0.117 (3)	−0.0004 (16)	0.023 (2)	0.011 (2)
C21	0.0449 (16)	0.050 (2)	0.068 (2)	−0.0045 (14)	0.0173 (15)	0.0152 (17)
C22	0.0261 (11)	0.0352 (15)	0.0313 (13)	−0.0037 (10)	0.0091 (10)	−0.0013 (11)
C23	0.0252 (11)	0.0253 (14)	0.0395 (14)	−0.0013 (10)	0.0041 (10)	0.0096 (11)
C24	0.0294 (12)	0.0518 (18)	0.0410 (15)	−0.0039 (12)	0.0059 (11)	0.0169 (14)
C25	0.0264 (12)	0.072 (2)	0.060 (2)	−0.0057 (14)	0.0089 (13)	0.0288 (18)
C26	0.0299 (14)	0.055 (2)	0.070 (2)	−0.0142 (14)	−0.0072 (14)	0.0151 (18)
C27	0.0420 (15)	0.0365 (18)	0.0589 (19)	−0.0057 (13)	−0.0041 (14)	−0.0049 (15)
C28	0.0309 (12)	0.0285 (15)	0.0465 (16)	−0.0027 (11)	0.0037 (11)	−0.0010 (12)

### Geometric parameters (Å, °)

**Table:**

Br1—C18	1.899 (3)	C14—C6^i^	1.515 (3)
Br2—C25	1.897 (3)	C14—H14A	0.9900
O1—C1	1.387 (3)	C14—H14B	0.9900
O1—C15	1.437 (3)	C15—C16	1.508 (4)
O2—C7	1.387 (3)	C15—H15A	0.9900
O2—C22	1.417 (3)	C15—H15B	0.9900
C1—C2	1.390 (3)	C16—C21	1.373 (4)
C1—C6	1.406 (3)	C16—C17	1.388 (4)
C2—C3	1.398 (3)	C17—C18	1.382 (4)
C2—C13	1.513 (3)	C17—H17	0.9500
C3—C4	1.376 (3)	C18—C19	1.373 (5)
C3—H3	0.9500	C19—C20	1.374 (6)
C4—C5	1.381 (4)	C19—H19	0.9500
C4—H4	0.9500	C20—C21	1.385 (5)
C5—C6	1.385 (3)	C20—H20	0.9500
C5—H5	0.9500	C21—H21	0.9500
C6—C14^i^	1.515 (3)	C22—C23	1.500 (3)
C7—C12	1.391 (3)	C22—H22A	0.9900
C7—C8	1.394 (3)	C22—H22B	0.9900
C8—C9	1.397 (3)	C23—C24	1.385 (3)
C8—C14	1.514 (4)	C23—C28	1.386 (4)
C9—C10	1.381 (4)	C24—C25	1.376 (4)
C9—H9	0.9500	C24—H24	0.9500
C10—C11	1.383 (4)	C25—C26	1.383 (5)
C10—H10	0.9500	C26—C27	1.367 (5)
C11—C12	1.394 (3)	C26—H26	0.9500
C11—H11	0.9500	C27—C28	1.389 (4)
C12—C13	1.516 (3)	C27—H27	0.9500
C13—H13A	0.9900	C28—H28	0.9500
C13—H13B	0.9900		
			
C1—O1—C15	115.49 (19)	H14A—C14—H14B	107.2
C7—O2—C22	113.41 (16)	O1—C15—C16	112.5 (2)
O1—C1—C2	118.64 (19)	O1—C15—H15A	109.1
O1—C1—C6	119.3 (2)	C16—C15—H15A	109.1
C2—C1—C6	121.7 (2)	O1—C15—H15B	109.1
C1—C2—C3	117.7 (2)	C16—C15—H15B	109.1
C1—C2—C13	122.5 (2)	H15A—C15—H15B	107.8
C3—C2—C13	119.8 (2)	C21—C16—C17	118.8 (3)
C4—C3—C2	121.5 (2)	C21—C16—C15	121.7 (3)
C4—C3—H3	119.2	C17—C16—C15	119.5 (2)
C2—C3—H3	119.2	C18—C17—C16	119.7 (3)
C3—C4—C5	119.4 (2)	C18—C17—H17	120.2
C3—C4—H4	120.3	C16—C17—H17	120.2
C5—C4—H4	120.3	C19—C18—C17	121.6 (3)
C4—C5—C6	121.5 (2)	C19—C18—Br1	119.7 (2)
C4—C5—H5	119.3	C17—C18—Br1	118.7 (2)
C6—C5—H5	119.3	C18—C19—C20	118.6 (3)
C5—C6—C1	117.9 (2)	C18—C19—H19	120.7
C5—C6—C14^i^	119.6 (2)	C20—C19—H19	120.7
C1—C6—C14^i^	122.5 (2)	C19—C20—C21	120.5 (3)
O2—C7—C12	118.30 (19)	C19—C20—H20	119.8
O2—C7—C8	118.9 (2)	C21—C20—H20	119.8
C12—C7—C8	122.7 (2)	C16—C21—C20	121.0 (3)
C7—C8—C9	117.6 (2)	C16—C21—H21	119.5
C7—C8—C14	122.9 (2)	C20—C21—H21	119.5
C9—C8—C14	119.4 (2)	O2—C22—C23	109.6 (2)
C10—C9—C8	120.8 (2)	O2—C22—H22A	109.8
C10—C9—H9	119.6	C23—C22—H22A	109.8
C8—C9—H9	119.6	O2—C22—H22B	109.8
C9—C10—C11	120.2 (2)	C23—C22—H22B	109.8
C9—C10—H10	119.9	H22A—C22—H22B	108.2
C11—C10—H10	119.9	C24—C23—C28	119.8 (2)
C10—C11—C12	120.9 (2)	C24—C23—C22	117.3 (2)
C10—C11—H11	119.5	C28—C23—C22	122.9 (2)
C12—C11—H11	119.5	C25—C24—C23	119.3 (3)
C7—C12—C11	117.7 (2)	C25—C24—H24	120.3
C7—C12—C13	121.6 (2)	C23—C24—H24	120.3
C11—C12—C13	120.7 (2)	C24—C25—C26	121.7 (3)
C2—C13—C12	111.12 (19)	C24—C25—Br2	117.7 (3)
C2—C13—H13A	109.4	C26—C25—Br2	120.7 (2)
C12—C13—H13A	109.4	C27—C26—C25	118.4 (3)
C2—C13—H13B	109.4	C27—C26—H26	120.8
C12—C13—H13B	109.4	C25—C26—H26	120.8
H13A—C13—H13B	108.0	C26—C27—C28	121.4 (3)
C8—C14—C6^i^	117.47 (19)	C26—C27—H27	119.3
C8—C14—H14A	107.9	C28—C27—H27	119.3
C6^i^—C14—H14A	107.9	C23—C28—C27	119.4 (3)
C8—C14—H14B	107.9	C23—C28—H28	120.3
C6^i^—C14—H14B	107.9	C27—C28—H28	120.3
			
C15—O1—C1—C2	−112.0 (2)	C1—C2—C13—C12	−105.0 (2)
C15—O1—C1—C6	74.0 (3)	C3—C2—C13—C12	71.8 (3)
O1—C1—C2—C3	−179.4 (2)	C7—C12—C13—C2	97.3 (2)
C6—C1—C2—C3	−5.6 (3)	C11—C12—C13—C2	−82.7 (3)
O1—C1—C2—C13	−2.6 (3)	C7—C8—C14—C6^i^	−44.1 (3)
C6—C1—C2—C13	171.2 (2)	C9—C8—C14—C6^i^	139.2 (2)
C1—C2—C3—C4	3.1 (4)	C1—O1—C15—C16	83.6 (3)
C13—C2—C3—C4	−173.8 (2)	O1—C15—C16—C21	−100.1 (3)
C2—C3—C4—C5	0.9 (4)	O1—C15—C16—C17	80.5 (3)
C3—C4—C5—C6	−2.6 (4)	C21—C16—C17—C18	−0.3 (4)
C4—C5—C6—C1	0.2 (3)	C15—C16—C17—C18	179.1 (2)
C4—C5—C6—C14^i^	177.7 (2)	C16—C17—C18—C19	0.8 (4)
O1—C1—C6—C5	177.8 (2)	C16—C17—C18—Br1	−177.41 (18)
C2—C1—C6—C5	4.0 (3)	C17—C18—C19—C20	−0.7 (5)
O1—C1—C6—C14^i^	0.3 (3)	Br1—C18—C19—C20	177.6 (3)
C2—C1—C6—C14^i^	−173.5 (2)	C18—C19—C20—C21	−0.1 (5)
C22—O2—C7—C12	95.5 (2)	C17—C16—C21—C20	−0.5 (4)
C22—O2—C7—C8	−86.3 (2)	C15—C16—C21—C20	−179.9 (3)
O2—C7—C8—C9	−177.8 (2)	C19—C20—C21—C16	0.6 (5)
C12—C7—C8—C9	0.4 (3)	C7—O2—C22—C23	−176.98 (19)
O2—C7—C8—C14	5.5 (3)	O2—C22—C23—C24	176.7 (2)
C12—C7—C8—C14	−176.3 (2)	O2—C22—C23—C28	−2.4 (3)
C7—C8—C9—C10	−1.4 (3)	C28—C23—C24—C25	0.3 (4)
C14—C8—C9—C10	175.5 (2)	C22—C23—C24—C25	−178.7 (3)
C8—C9—C10—C11	0.8 (4)	C23—C24—C25—C26	0.5 (5)
C9—C10—C11—C12	0.8 (4)	C23—C24—C25—Br2	−180.0 (2)
O2—C7—C12—C11	179.3 (2)	C24—C25—C26—C27	−0.5 (5)
C8—C7—C12—C11	1.1 (3)	Br2—C25—C26—C27	180.0 (2)
O2—C7—C12—C13	−0.8 (3)	C25—C26—C27—C28	−0.3 (5)
C8—C7—C12—C13	−179.0 (2)	C24—C23—C28—C27	−1.1 (4)
C10—C11—C12—C7	−1.7 (3)	C22—C23—C28—C27	177.9 (3)
C10—C11—C12—C13	178.3 (2)	C26—C27—C28—C23	1.1 (4)

Symmetry codes: (i) −*x*, −*y*+2, −*z*+1.

### Hydrogen-bond geometry (Å, °)

**Table:**

Cg1 and Cg2 are the centroids of the C23–C28 and C7–C12 rings, respectively.

**Table:**

*D*—H···*A*	*D*—H	H···*A*	*D*···*A*	*D*—H···*A*
C15—H15B···Br2^ii^	0.99	2.91	3.652 (2)	132
C17—H17···Cg1	0.95	2.74	3.69	175
C5—H5···Cg2^iii^	0.95	2.68	3.54	151

Symmetry codes: (ii) −*x*+1, −*y*+2, −*z*+1; (iii) *x*, −*y*+3/2, *z*+1/2.
